# Tissue-Derived Stem Cell Research

**DOI:** 10.1155/2016/4801953

**Published:** 2016-01-17

**Authors:** Ming Li, Kequan Guo, Dong Hyun Kim, Luca Vanella

**Affiliations:** ^1^Department of Stem Cell Disorders, Kansai Medical University, Hirakata, Osaka 5731010, Japan; ^2^Department of Cardiac Surgery, Beijing Institute of Heart, Lung & Blood Vessel Disease, Beijing Anzhen Hospital, Capital Medical University, Beijing 100029, China; ^3^Northwest Ohio Orthopedics and Sports Medicine Inc., NWO Stem Cure, LLC, 7595 County Road 236, Findlay, OH, USA; ^4^Department of Drug Science, Section of Biochemistry, University of Catania, 95125 Catania, Italy

Tissue-derived stem cells (TDSCs) are undifferentiated cells, presented in tissues such as bone marrow, blood vessels, and adipose tissues and with the ability to repair damaged areas by generation of new cells and tissues. TDSCs have proven to be a feasible source of cells for tissue regeneration medicine in recent experimental and clinical studies. Mesenchymal stem cells revealed potential benefits in atherosclerosis [1] and endothelial progenitor cells reduced lung damage and improved lung function [2]. Moreover, there were some reports that suggested that adipose derived stem cells may improve injured lung function [3], infracted heart function [4], and injured kidney and its function [5]. Regulation of the process to successfully trigger proper differentiation into the desired cell types is important. Moreover, it is more important to confirm safety of transplantation when stem cells are used to treat diseases.

In the present special issue, the significance and possible clinical applications of TDSCs were presented. Some manuscripts described important biochemical cascade underlying the processes of osteogenesis, adipogenesis, angiogenesis, and their possible applications in new therapy development. All findings and experiences of TDSCs research will open up new possibilities for the treatment of various diseases and extend the human life span.

*Ming Li* *Ming Li* *Kequan Guo* *Kequan Guo* *Dong Hyun Kim* *Dong Hyun Kim* *Luca Vanella* *Luca Vanella*
